# Hands-Free User Interface for AR/VR Devices Exploiting Wearer’s Facial Gestures Using Unsupervised Deep Learning

**DOI:** 10.3390/s19204441

**Published:** 2019-10-14

**Authors:** Jaekwang Cha, Jinhyuk Kim, Shiho Kim

**Affiliations:** Seamless Transportation Lab (STL), School of Integrated Technology, and Yonsei Institute of Convergence Technology, Yonsei University, Incheon 21983, Korea; chajae42@yonsei.ac.kr (J.C.); jinhyuk.kim@yonsei.ac.kr (J.K.)

**Keywords:** hands-free interface, augmented reality, spatiotemporal autoencoder, deep embedded clustering

## Abstract

Developing a user interface (UI) suitable for headset environments is one of the challenges in the field of augmented reality (AR) technologies. This study proposes a hands-free UI for an AR headset that exploits facial gestures of the wearer to recognize user intentions. The facial gestures of the headset wearer are detected by a custom-designed sensor that detects skin deformation based on infrared diffusion characteristics of human skin. We designed a deep neural network classifier to determine the user’s intended gestures from skin-deformation data, which are exploited as user input commands for the proposed UI system. The proposed classifier is composed of a spatiotemporal autoencoder and deep embedded clustering algorithm, trained in an unsupervised manner. The UI device was embedded in a commercial AR headset, and several experiments were performed on the online sensor data to verify operation of the device. We achieved implementation of a hands-free UI for an AR headset with average accuracy of 95.4% user-command recognition, as determined through tests by participants.

## 1. Introduction

**Figure 1:**
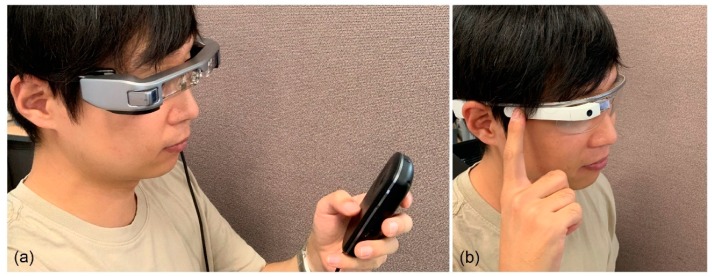
Example of common interaction method with a user wearing an augmented reality (AR) headset: (**a**) hand-held controller and (**b**) button or touchpad.

Augmented reality (AR) is one of the hottest issues in the information and communication technology (ICT) industry. In the field of AR, developing a user interface (UI) device that is optimized for AR headsets is one of the key challenges. This is because the headset environment, which differs greatly from a personal computer or mobile phone, makes it difficult to use conventional UIs (e.g., keyboard, mouse, touch screen, etc.). Pressing buttons on the side of the headset or holding remote controllers are the most typical solutions currently adopted for headset UIs. However, such UIs not only have limitations in supporting complex and dangerous operations, such as surgery or manual labor, but are also insufficiently convenient for general usage. Voice recognition techniques and physiological sensors, such as electrooculography (EOG), electroencephalography (EEG), or electromyography (EMG), are promising alternative means of providing a hands-free UI for AR headsets, however, optimal sensing devices and methods for implementation of headset UI have yet to be developed. Figure 1 depicts an off-the-shelf AR headset user using a hand-held controller or buttons (or touchpad) to interact with the headset system.

To address this unsolved requirement for a hands-free natural user interface (NUI) for AR/VR devices, we propose a method to detect skin deformation using a custom-made sensor. The purpose of the proposed technology is to provide a hands-free UI system for an AR headset, which exploits the facial gestures of users as the input commands of the UI. The proposed technique facilitates the detection of facial skin movements in a noninvasive manner and supports hands-free interaction between the headset user and the AR system by mapping a detected facial gesture of a user to an input command.

The custom-made sensor monitors the deformation of the facial skin of headset users and uses the facial-skin movement to recognize the facial gestures of the user. The skin deformation is detected by measuring spatially resolved diffuse reflectance (SRDR) [1,2,3,4] of near infrared (NIR) light on human skin. The sensor includes an NIR laser diode (LD) and an IR camera. The NIR laser is focused on the region of interest (ROI) on the skin, and the camera tracks changes of IR SRDR on the skin by capturing sequential image frames of SRDR. Changes in SRDR give clues allowing detection of the facial gestures because the captured sensor data vary depending on skin deformation conditions [5].

Tracking predefined feature points on the human face is a widely used method for facial-gesture detection [6]. In this context, the proposed method classifies the skin deformation data detected from the ROI of the sensor in order to recognize the facial gestures. The SRDR data, which indicate the skin deformation, were collected in the form of image sequences and contain spatiotemporal information about the skin deformation. A skin deformation data classifier was designed based on the deep neural network (DNN) technique to extract and utilize the spatiotemporal features of the input data. We adopted spatiotemporal autoencoder (STAE) [7] and deep embedded clustering (DEC) [8] algorithm to implement the classifier.

STAE is frequently exploited for studying video anomaly detection [9,10,11,12] and is an application for finding outlier patterns in a video. It is popular because this network shows remarkable performance for learning the representation of sequential images that contain both spatial and temporal information. The STAE extracts spatiotemporal features in the sequential NIR images of the proposed sensor module without a human in-the-loop for supervision. DEC boosts the performance of the K-means clustering algorithm [13] by fine-tuning the pretrained weights of the STAE for separation of the cluster distribution. DEC could be combined with various other autoencoders, such as a stacked autoencoder (SAE) [8] or a fully convolutional autoencoder (FCAE) [14].

The proposed network was trained using the sequential images of the NIR SRDR dataset collected from experimental volunteers. We installed the proposed UI device on an Epson BT-350 AR headset and then implemented an AR headset UI based on the proposed device. Several experiments were conducted for verification of the operation of the proposed UI. The contributions of our work can be summarized as follows:

- Implementation of an IR sensor for skin-deformation detection;
- Design of a classifier neural network for spatiotemporal data processing;
- Conception and realization of a hands-free UI for AR headsets.

The remainder of this paper is organized as follows. In Section 2, we describe prior work related to this research. Section 3 explains the working principles of our facial-gesture recognition sensor module and our implementation of the sensor. In Section 4, we present how to implement the sensor data classifier network. The results of the UI implementation and discussions are reported in Section 5 and Section 6, respectively. Conclusions of this paper are provided in Section 7.

## 2. Related works

### 2.1. UI for Headset Environments

Many researchers in academia or industry have already developed advanced devices and solutions enabling user interaction with headset-type devices. Hand gesture detection or eye-gaze tracking technologies are some of most popular methods for this purpose. Using eye gaze to control an AR system was tried [15]. Those researchers designed a pupil and gaze estimation chip and proposed an object recognition system for AR headsets. In [16], a two-dimensional gesture interface for AR glasses was proposed. The interface system received hand or foot gestures as input commands. In another case, a commercial electromyography (EMG)-based interface sensor (MYO) was adopted to build a hand-gesture-based interface [17]. Gugenheimer et al. introduced a touch UI for a mobile virtual reality (VR) headset named FaceTouch [18]. A touchpad was attached to the backside of a commercial VR headset to provide touch UI. However, the interaction methods of most commercial AR headsets are still based on peripheral hand-held controllers or buttons on the headset [19,20] because the alternative technology needed for more effective UIs has not matured enough.

### 2.2. Facial Gesture Recognition for Headset Environments

#### 2.2.1. Camera-Based Approaches

Facial gesture recognition technology is a great alternative solution for developing UI for headset device environments. The best-known method for the facial gesture recognition is facial image processing with an external camera. Liu et al. presented a boosted deep belief network (BDBN) for the facial expression recognition from facial pictures [21]. Their network was able to recognize six emotions that appeared in the pictures. For headset-type devices, however, this method is hard to apply as an input device for a UI. Because a headset covers a significant portion of the face, the acquisition of proper facial images is prevented. To overcome this problem, Khan et al. introduced an upper face (near-eye) region reconstruction technique with a lower face (near-mouth) picture and an eye picture taken from the side [22]. Their technique was based on an asymmetrical principal component analysis (aPCA) framework. Using a camera inside the device is an alternative method to take photographs of facial images. However, the restricted distance from the built-in camera lens to the facial skin made the fields of view insufficient to capture the significant facial regions. Olszewski et al. installed cameras near eyepieces of their VR headset [23]. Their system took pictures of near-eye and near-mouth regions, respectively, to find features for facial expression recognition. They demonstrated the facial expression recognition results by representing them on a high-fidelity 3D avatar. Hickson et al. tried to classify facial expression with only near-eye images taken by eye-tracking cameras in the headset [24]. They achieved 74% recognition accuracy among five facial expressions.

#### 2.2.2. Contact-Based Approaches

McFarland et al. used an electroencephalogram (EEG) to sense brain activities and facial expressions by recording brain waves for brain–computer interfaces and control [25]. This method, however, requires many learning sequences showing user concentration during measurement. Piezoelectric sensors hidden in a visor extension of smart glasses were also used for detecting facial expressions [26]. This device enables the recognition of some kinds of expression, except static states, such as keeping the mouth open. An EMG sensor catches the electrical activity caused by the movement of motor units in the skeletal muscles. The researchers in [27] modeled the speech motor system of a human face by observing the movement of muscles, using EMG sensors. The quality of EMG signals, however, commonly depends on the location of the sensor attachment, and the signals are prone to crosstalk due to other motor units, as well as noise due to the resistance between sensors and the contact surface. Other authors [28] proposed a facial expression recognition headset in which eight strain gauge sensors were mounted on the foam liner of the headset for the upper face. The sensors detected expressions involving the upper face, and an additional camera mounted on the bottom of the headset tracked expressions of the lower face. Strain gauge sensors, however, still have an inherent limitation for precise tracking of locally stretched or compressed (deformed) human facial skin. This is because of the curved 3D shape of the peripheral region of the eyes and nose. Moreover, these kinds of sensors are sensitive to the quality of the sensor-skin contact state. This makes them unsuitable for an AR headset environment, which has less skin contact area and accompanies frequent facial motion, which would degrade the state of sensor contact with the skin.

#### 2.2.3. Optical-Based Approaches

An optical-based sensor exploits infrared (IR) reflection or diffusion characteristics for recognition of facial gestures. Masai et al. integrated photo-reflective sensors on the inner surface of a glasses frame to recognize the user ’s facial expression [29]. The array of sensors measured the distance to the facial skin surface and exploited the data as the features of facial expressions. Suzuki et al. also integrated a sensor into their VR headset frame and utilized the mentioned sensor to recognize facial expressions of a VR headset user [30]. This kind of photo-reflective sensor has a small form-factor and works in contactless mode, which can be helpful for eliminating drawbacks of a contact-type sensor. However, the person-to-person or experiment-to-experiment variation of the operating distance may cause fluctuations and sensing error.

## 3. Sensor Implementation

### 3.1. IR Diffusion in Human Skin and Skin Deformation

Deformation of human skin can be detected by measuring changes in the SRDR of IR transmitted underneath the skin. “Diffuse reflectance” (DR) is the ratio of the amount of flux reradiated by diffuse reflection, and its intensity is affected by the microstructure of collagen fibers distributed under the skin. “Spatially resolved” (SR) implies using the radial distance from a light incident spot [2,4,31]. Nickel et al. found that “Langer ’s line” [32], which indicates the collagen fiber distribution in human skin, follows the direction of measured SRDR of skin [4], and Cha et al. showed that the SRDR measured in relation to skin deformation mirrors deformation of the skin [5]. Cha et al. and Kim et al. applied IR diffusion reflectance to detect VR headset user’s facial expressions [33,34].

### 3.2. Target Gesture Selection for the UI Implementation

One of our goals in this study was to implement a general-purpose UI device as reliable as a mouse (as widely used with personal computers) for the AR headset environment by utilizing facial gestures combined with head movements. Similar to the basis of a mouse UI, the proposed headset UI consists of input motions for moving a cursor and clicking a pointer on the display. By combining intentional facial wink gestures and user head movements, we could implement user input commands for an AR headset. An on-board gyro sensor detects rotational movement of a user’s head and the proposed IR sensor module determines the intention of the user to click pointers. The author’s design principle of the proposed UI is to make inputting the command-set of an AR headset as simple as using a mouse with personal computers. We only detected simple wink gestures, even though it is not difficult to detect subtle expressions of the user, because too many subtle gestures may increase the complexity of the user interface to the extent that it becomes inconvenient as a UI.

### 3.3. Prototyping the Sensor Module

**Figure 2:**
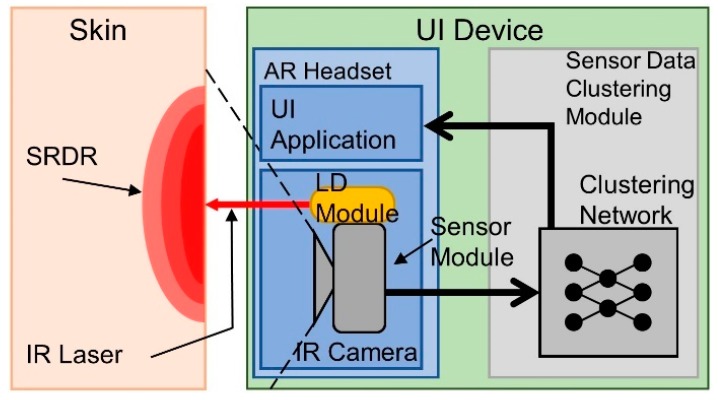
Overall system diagram: The sensor module includes an IR LD and an IR camera. The IR camera takes images of IR diffusion patterns, and the LD emits IR light onto the skin.

The proposed hands-free UI system included a sensor module that detected IR diffusion patterns from the sensor wearer ’s skin surface and a sensor data classifier based on a DNN structure. Figure 2 depicts the overall configuration of the proposed system. The sensor module was installed on a wearable AR headset. The module consists of an LD and a USB camera as a pair: IR emitter and receiver. While the user was wearing AR glasses, the IR light emitted from the LD was diffused through the user ’s skin. The camera captured sequential images of the diffused IR light and sent the images to the classifier to convert the user ’s skin deformation state to an interface command. We fabricated a prototype by using a commercial AR headset (Epson BT-350) with an in-house-made facial-gesture tracking sensor module. The sensor module was installed on the left side of the glasses, and the ROI sensor was aimed at a spot between the user ’s left eye and ear, where the skin is locally stretched or compressed when a user makes winking gestures. The NIR LD (i.e., IR emitter) had an 850 nm centroid wavelength with 0.4 mW average output power and 65 mrad beam divergence with 2.5 mm laser module aperture diameter. According to ANSI Z136.1-2014 laser safety standard [35], output power density below 2.02 mW/cm^2^ at human pupil is categorized as a class 1 laser, which is considered to be safe under all conditions of normal use for NIR laser. The output power of our laser emitter is 0.4 mW, which is much smaller than the acceptable standard. Further, we have used the 10% transmittance filter in front of our emitter module to get ten times lesser output power from the emitter to guaranty the safety of users. The NIR illumination did not interfere with the user ’s visual field, and we limited the NIR dose to a safe level to prevent any potential harmful effects to the human eye.

**Figure 3:**
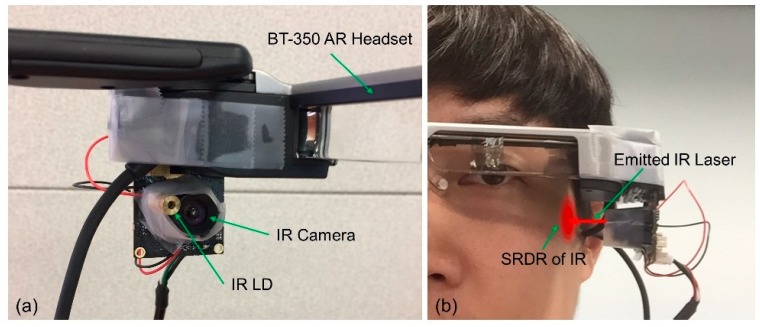
Implementation of the sensor module. (**a**) The sensor module includes a USB camera and NIR laser diode. We installed the module on the left side of the Epson BT-350 AR glasses. (**b**) A photograph of the headset on a user. The laser diode is aimed on the skin near the left cheek, which is deformed when a user makes winking gestures.

The NIR band has the deepest skin penetration depth of irradiation in the harmless light spectrum [36]. This is an important property in the selection of the light source for implementation of the sensor, coupled with its low power consumption. We chose a laser diode (LD) instead of a light emitting diode (LED) as an IR light source, even though the LED is more durable in practice. This is because IR light from an LED has insufficient collimation to be transmitted through human skin when LED and skin are not in direct contact, in contrast to the light of an LD. The IR emitter included an LD, a protection circuit, and a collimator lens. The protection circuit is to extend the lifespan of the LD, and the lens collimates the IR laser beam to reduce divergence of the emission. The IR receiver is a sensor module containing an OV2710 chipset camera. This has an IR band pass filter to detect IR light without confounding the effects arising from ambient visible light. The field of view (FOV) of the camera is 100 degrees, and it records at 120 fps with 320 × 240 resolution, according to the manufacturer’s specifications. In our system, the camera ran at (on average) 30 fps because of the data processing latency. In practice, we used a 320 × 240 resolution grayscale jpeg image for the input data. Figure 3 shows the results of the sensor module implementation on the AR headset.

**Figure 4:**
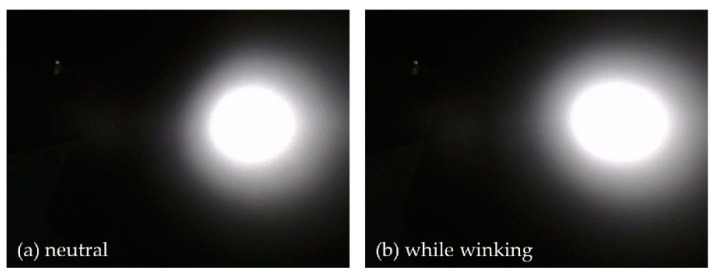
Images of IR laterally propagated through the skin: (**a**) IR SRDR image captured with no facial gesture and (**b**) captured IR SRDR image during a wink gesture by the user. The brightness of the white region in these images represents the intensity of the IR SRDR.

Figure 4 shows some IR diffusion patterns recorded by the sensor module while the user was wearing the AR headset. The NIR band pass filter installed in the camera cut all wavebands of light except 850 nm (NIR). The shape of the pattern changed in relation to the facial gestures of the user.

## 4. Design of Sensing Data Classifier

### 4.1. Classifier Design and Dataset Collection

As illustrated in the previous section, the data input to the data classifier was in the form of NIR SRDRs images taken from human skin. The camera-captured SRDR shape may have person-to-person variations due to many hardly modeled factors. The proposed sensor data classifier had to detect the transition of the captured SRDR shape to recognize the user’s gesture. We utilized unsupervised learning methods for the neural network sensor data classifier. The unsupervised learning methods provided us an easy way to calibrate when we had to cope with person-to-person variation, because we could avoid data annotation tasks for generation of the training dataset.

The process for training the sensor data classifier is composed of two phases:

1. Unsupervised learning for extracting features from the dataset using an autoencoder;
2. Clustering and fine-tuning the extracted features to boost the performance of the classifier.

We used the in-house IR sensor module shown in Figure 3 to collect training data for the classifier neural network. We collected more than 5000 sequential SRDR images per person, without data annotation for the training dataset of the proposed classifier network. In this paper, we used only 81,758 SRDR images from 10 different people for training the classifier network.

### 4.2. Preprocessing of SRDR Image Data

**Figure 5:**
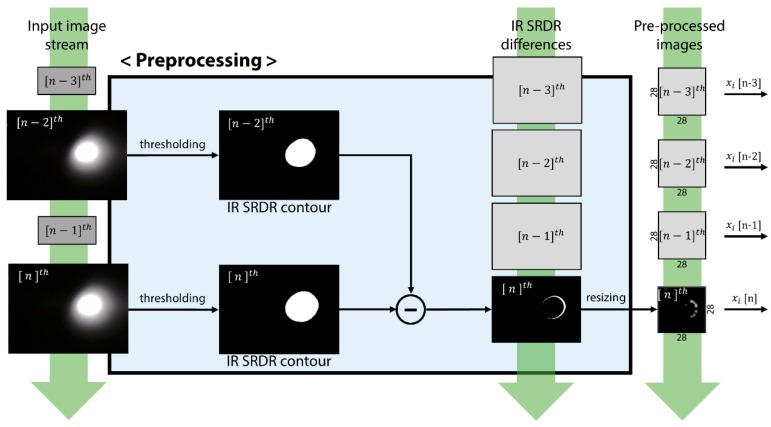
Preprocessing procedure of the clustering network: The preprocessing unit calculates the difference between two images. For the calculation, the unit thresholds the input images, conducts pixel-wise subtraction between the images, and resizes the images to 28 × 28 pixels to suit the clustering network input size.

Data input to the classifier network were a series of four 320 × 240 pixel IR diffusion snapshot images, which were taken sequentially by the camera and stored in an input image queue. The network performed preprocessing before sending the images to the feature extraction part of the network. In the preprocessing procedure, the preprocessing unit highlighted the transition of the SRDR contour shape to make the classifier network focus on variation of the SRDR contour shape, and then grouped the four sequential images to meet the RNN window size of the STAE. The unit first converted the input image to a black-and-white binary image with an empirically determined threshold value to highlight the contour shape of each SRDR. In addition, the threshold process reduced the sensor noise due to unwanted external IR light sources, such as the sun or fluorescent lamps. Then, the unit calculated pixel-wise differences in the SRDR binary images between certain time steps (which means two sampling intervals between the present sample time (n) and the previous time (n-2), in practice) to highlight the variation of the SRDR contour shape. After that, the images were resized into 28 × 28 pixel images to save computational resources. The past images were stored in the image queue. Finally, the unit composed four sequential images as a set of input image sequences for input to the feature-extraction part. The image at the end of the input sequence indicated the latest image captured from the camera. The explained preprocessing procedure is illustrated in Figure 5.

### 4.3. STAE for the Spatiotemporal Feature Extraction

The camera of the proposed sensor converted user facial gestures into sequential NIR SRDR images. Those images were the data input to the classifier network, which decided whether the data implied a winking gesture. Image clustering is a promising method for image classification in an unsupervised way; however, this is still a hard task for the following two reasons. The first issue is the dimensionality of the input image data. Images are often of very high dimensionality, and this should be reduced to avoid the “curse of dimensionality” [37], which significantly affects the performance of a K-means algorithm. Another issue is that features in the images usually have 2D or 3D local structures, which should not be overlooked during the clustering. These features require kernels optimized to the shape of the local structures. Moreover, because the inputs from the sensor were sequential data, which may have temporal features, the proposed clustering network should be able to extract temporal features, as well as spatial information, from the inputs.

We adopted STAE [7] to reduce the input dimensions, while preserving the spatiotemporal features of the input without human supervision. Using the encoder part of an autoencoder is a widely used technique for building a feature extractor as a clustering method. This is because the encoder learns how to compress the input data with minimization of the compression loss while training the autoencoder. Moreover, as the encoder was trained based on autoencoder network, the encoder prevents the salt and pepper noise that may remain after the preprocess of the input image. STAE is a type of autoencoder composed of a convolutional neural network (CNN) and a recurrent neural network (RNN) for the extraction of spatial and temporal features from the input data, respectively. For the case of clustering n image sequences ${\left\{x_{i}\in X\right\}}_{i=1}^{n}$ into *k* clusters represented by a centroid $\mu_{j}$, *j* = 1, *…*, *k*, STAE was able to reduce the dimensionality of each $x_{i}$ with nonlinear mapping $f_{\theta }\;:X\;\to Z$, where θ is the weight of the kernels and Z is the latent feature space. The kernels not only support the dimensional reduction, but also have the capacity to detect features in the sequences of images. At the end of STAE learning, the training θ is completed (which implies initialization of the classifier parameter), and the decoder part of the STAE is no longer needed. It is then discarded in the subsequent boosting step.

### 4.4. Designing and Training of the Feature Extractor

**Figure 6:**
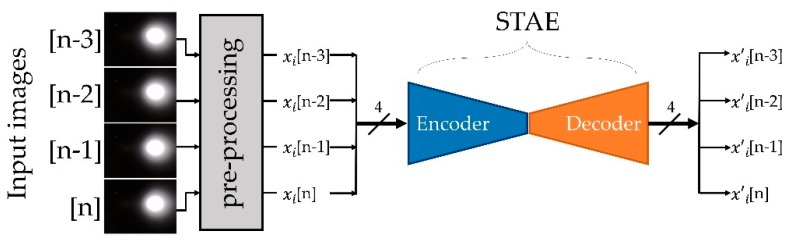
Structure of the network extracting sensor data features: spatiotemporal autoencoder (STAE) consists of an encoder and a decoder. After the training of STAE, only the encoder part is utilized as the feature extractor.

The autoencoder is a type of artificial neural network that learns the data representation in an unsupervised manner and typically is part of an encoder–decoder pair. The encoder is trained to compress the input data, while the decoder is trained to restore the compressed data to the original input data with minimum loss. In this context, the encoder was induced to learn data representations to reduce redundancy in the input data. As illustrated in Figure 6, the proposed STAE received four successive images as inputs from the preprocessing unit.

**Figure 7:**
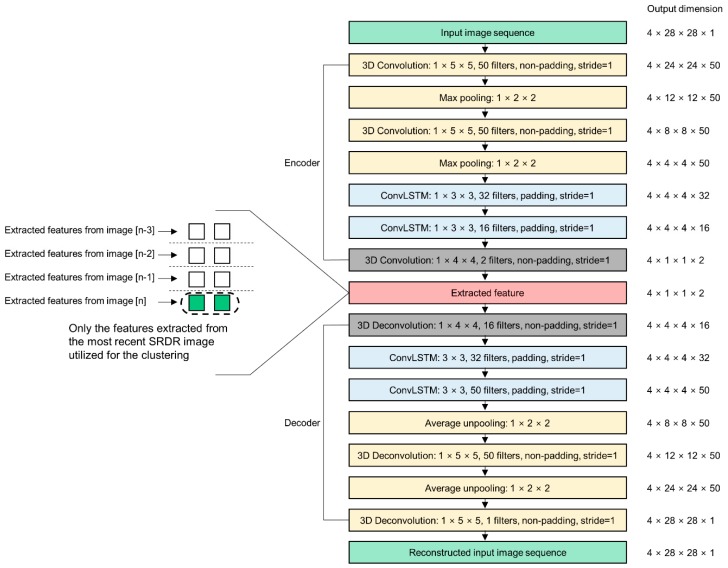
Detailed configurations of the STAE used for feature extraction.

Figure 7 depicts details of the network structure of the proposed STAE. To find spatial features and to reduce the input image size, two 3D CNN layers were arranged foremost in the proposed STAE network. To reduce the dimensionality of the input images, each convolution layer of the encoder operated in non-padding mode. For initialization of the kernel weights, variance scaling [38] was used. Each CNN layer was followed by a maximum pooling layer that reduced the image size by half. Because the input data was 3D (width, height, and sampled time), the network required not a general 2D CNN, but 3D CNN layers, to process the input data. Then, two successive convolutional long-short-term memory (ConvLSTM) [39] layers extracted temporal features from the compressed input data. Traditional RNN is able to learn temporal features with hidden state parameters; however, it has the vanishing gradients problem [40]. Long-short-term memory (LSTM) alleviates the vanishing gradients problem by introducing a recurrent gate called a forget gate [40]. ConvLSTM is a kind of LSTM designed to find temporal information from a sequence of image frames, such as video data and has 2D shape weights. Instead of using a multiply operation between the inputs and weights, ConvLSTM uses a convolution operation between them. This LSTM layer has the ability to propagate the spatiotemporal features through each LSTM state. Finally, we arranged another 3D CNN layer behind the ConvLSTM layers, which was designed to have a kernel size identical to the compressed input images, to convert the four 2D images into four 2D vectors that implied the extracted features. This layer mapped the outputs of the former layer to 2D space for clustering. We empirically set the dimensions of the feature space to match that of the intended number of clusters. Because the number of clusters we wanted to make involved only two states (skin deformation state and no-change state), we set the dimensions of the feature space to 2D. Only the last vector, which implies the extracted feature of the latest captured data, of the four extracted vectors was exploited in the sensor data clustering procedure as a final result of feature extraction. The configuration of the decoder was completely symmetrical to that of the encoder except for adopting the average unpooling layer instead of using the maximum unpooling layer for the convenience of network implementation. After learning for the autoencoder, the decoder was discarded from the classifier network because it was no longer needed. Only the encoder part was used in the feature extractor.

### 4.5. Sensor Data Feature Clustering and the Classifier Network Fine-Tuning with DEC

Clustering is one of the most commonly used unsupervised learning techniques, and it is used to group data that have similar characteristics. Compared to supervised learning-based classification, clustering has the advantage of not needing a background answer for the data. For the implementation of clustering, many researchers have adopted the K-means algorithm [13] because it is simple and relatively efficient compared to other clustering algorithms. However, K-means algorithm-based unsupervised clustering shows lower accuracy than that of supervised classification methods, in general.

The sensor adopted deep embedded clustering (DEC) method, which was introduced by Xie et al. [8], to reinforce clustering network performance. The proposed clustering method has a two-step strategy to enhance clustering quality. The first step is to find the initial cluster centers in the sensor data feature space by just applying the K-means algorithm to them. The second step is to apply a fine-tuning technique (i.e., DEC) to the overall classifier network, recurrently, in order to increase the accuracy until the refinement action ceases to provide more effectiveness. During the deep embedded clustering process, outliers or noise data points of clustering are suppressed [8,14].

**Figure 8:**
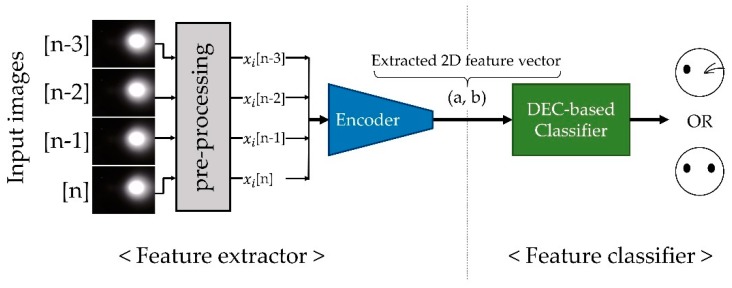
Proposed classifier network consisting of a STAE-based feature extractor and a deep embedded clustering (DEC)-based feature classifier.

DEC clusters data by simultaneously refining a pretrained set of k cluster centroids ${\left\{\mu_{j}\in Z\right\}}_{j=1}^{k}$ in feature space Z and the weights θ of STAE that encode data for input to Z. The centroids $\mu_{j}$ and weights of the encoder θ, are calculated iteratively during the DEC procedure until the parameters show optimal clustering results. We calculated the soft assignment of the original distribution *Q* and the target distribution *P* using the functions mentioned in [8] as the first step. Then, we obtained the difference between those two distributions using KL divergence, which is usually utilized to obtain the difference between two distributions. The calculated difference is used as a loss function of DEC for the network training. It was intended to improve the performance of the classifier by backpropagating the loss and updating θ and μ. This procedure was reiterated until the hard assignments from *Q* remained unchanged within a predefined threshold value. After the fine-tuning process, the weights of the encoder and centroid coordinates of the clusters were optimized for the classification of the input image stream. Figure 8 shows the overall structure of the proposed classifier network.

## 5. Results

**Figure 9:**
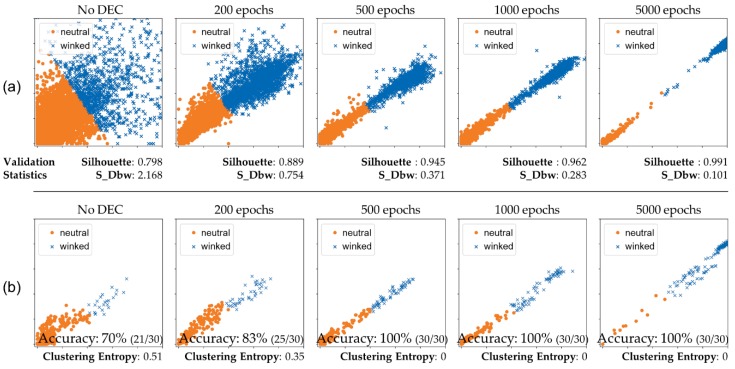
Clustering results by applying proposed DEC method: (**a**) clustering results for 81,758 training dataset images and (**b**) clustering results for real-time sensing from users (online validation).

Figure 9a shows the clustered results of the proposed classifier network with the number of iterations with the DEC method. The network clustered a sequence of 81,758 training-set input images to verify the performance of the clustering process. Because the feature space of the classifier was two-dimensional, the clustering results were able to be represented in 2D feature space without any dimensional reduction visualization method such as t-SNE [41]. Colors and shapes of the dots indicate each cluster of neutral or winked states, and the name of the cluster is depicted in the legend of the scatter plots. Compared to the results obtained before DEC, the results after application of the DEC significantly improved the resolution of the cluster boundary as the learning epochs proceeded. However, we could not determine the accuracy of the clustering network because we used unannotated data for the feature extraction and DEC-based classifier. We applied an unsupervised learning method to the proposed classifier network, so there was no information to indicate whether a state was winked or neutral.

Instead of conducting clustering validation with label information, we first validated the quality of cluster results by utilizing internal clustering validation statistics. For the validation, we calculated the silhouette coefficient (the larger, the better) and S-Dbw (the smaller, the better), which were promising internal clustering validation statistics reported in [42], and the results were shown in the bottom of Figure 9a. The result indicates the quality of clustering is enhanced by the increasing number of training epochs.

**Figure 10:**
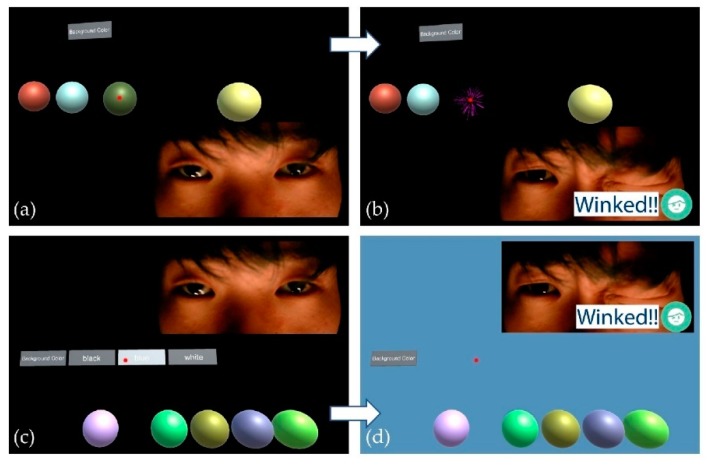
Screenshots from the demonstration using a custom-made application. A user could pop balloons (**a**,**b**) or select buttons to change the background (**c**,**d**). A user could select an object by aiming (targeting) a red center dot on a specific object and then execute using a winking gesture.

For evaluation of the accuracy of the proposed classifier network, we conducted additional experiments (for a second experimental dataset) with users wearing the proposed AR headset. Because we had not collected datasets for validation and testing, we utilized a person’s online sensor data as a validation dataset. In the experimental validation step, we applied the trained weights to the network, depending on the number of iterations of learning, and then measured the classification accuracy. Figure 9b shows the real-time clustering result for the online sensor data obtained from the additional experiments. Each experimental participant winked 30 times during each measurement, and we recorded the number of successful detections of an intentional wink gesture. We also calculated the entropies [43] of clustering results and summarized them in the bottom of Figure 9b. Figure 9b shows that performance of the clustering network improved as the epoch of the learning steps increased. After 500 iterations of the DEC learning, the network achieved 30/30 accuracy for online wink-gesture detection and reached entropy value of zero. The proposed gesture detection system operated in real-time mode at a speed of 30 fps. To test whether this classifier network could be applied to other people who had not participated as volunteers in gathering data, we performed a third set of additional experiments, shown in the screenshots from the demonstration, using a custom-made application. A user could pop balloons (Figure 10a,b in discussion section) or select buttons to change the background (Figure 10c,d in discussion section). A user could select an object by aiming at (targeting) a red central dot on a specific object and then execute using a winking gesture.

In the third set of experiments, participants were able to see the results of the gesture detection in response to their facial gestures in real time, and when the wink gesture was detected by the sensor, they received a score of one point. Each participant needed to earn 100 points to complete her or his experiment. We counted the number of intended wink gestures made by each participant until completing each experiment. These experiments were conducted using ten people who were all fresh participants (i.e., who had not participated in the training dataset collection). The proposed wink-gesture classifier achieved 95.4% average accuracy, with the worst case of entropy of 0.24, in regard to the ten participants listed in Table 1.

## 6. Discussion

Even though the proposed method utilized an IR camera as an input device, it was completely different from conventional image-based classifications of facial expressions. The proposed sensor detects the movement of muscles underneath the skin surface by tracking the distorted alignment of collagen fiber in the skin based on IR diffuse reflectance. It detects the skin deformation of the ROI without contact and does not require whole facial images. Because humans normally blink and wink intentionally, using different facial muscles, the proposed SRDR sensor can distinguish the two actions. Another strong point of this sensing method is that the proposed sensor could generate classification features from a plain surface of facial skin where no features could be found by camera.

We achieved the clustering of IR diffusion images generated by AR headset-user winking gestures with a custom-made facial gesture detecting sensor and a classifier network. The proposed sensor provides an efficient way to implement a UI for the AR headset. Many commands could be applicable with a combination of facial gestures and head rotation data, the latter of which are captured by the gyro sensor commonly integrated in most AR headsets. For example, the user could select a button by winking, or the user could move some objects in virtual space by rotating the head and manipulating it with a wink gesture, analogous to the drag-and-drop command commonly used in the desktop environment. Figure 10 shows screenshots taken from the in-house-made AR application using the proposed UI. Users could pop balloons or select a button in the AR environment by making a winking gesture. The authors believe that the proposed system may provide a user-friendly UI with the advantages of simple, hands-free control and low-cost of implementation.

Our training database consists of two facial gestures (neutral and wink) from 10 people between 23 to 36 years old. Our training database is limited, so 70% of the subjects were male, and the training dataset was generated from Korean people. However, we had verified that this interface device operates quite well regardless of the ethnic group of users in the demonstration (illustrated in Figure 10), with more than 100 people worldwide [44]. The demonstration results made us confident that the demographic properties may not seriously affect the tendency of IR SRDR contour changes triggered by facial gestures.

The sensors require appropriate positioning and are limited to certain locations on the human body to detect facial skin deformation. This is because the amount of change in the IR SRDR is sufficient only where the muscles beneath the skin’s surface move sufficiently to influence the internal structure of the skin. For the selection of the sensor detection spot, a facial action coding system (FACS) [45] should be considered. FACS deconstructs facial expressions of humans into specific action units (AUs), which are closely related to the muscular movement. For example, the wink gesture could be represented by AU 6, which is related to orbicularis oculi muscle. To achieve the best result, it is critical to set an appropriate ROI for the sensor. Inappropriate positioning of a sensor causes it to generate false negative errors. In addition, winking could be a tiresome or even difficult motion for some people. However, the intended scope of the work reported in this paper was to show the feasibility of the proposed facial gesture detection method and to implement a prototype sensor.

We focused on building a simple, robust, and cost-effective mouse-like, hands-free interface for AR headsets. As shown in the application demonstration in Figure 10, the proposed interface provides cursor-moving and cursor-clicking manners to achieve the intention. We can detect other kinds of facial gestures by selecting other sensor detection locations and increase the number of detectable facial gestures by adding more sensors for more-complicated and more-flexible system applications. It is expected that we may find the best detectable gestures and the optimal number of sensors for implementing a UI system through usability tests in future work. Developing the AR-based assistance in various demanding fields, such as medical applications (like during surgery, etc.), automobile industries (like the labor assistance, etc.), or extreme sports (like motorcycle-driving assistance, etc.) offers promising future work for this technique.

## 7. Conclusions

We developed a system that provides a hands-free UI for an AR headset, which consists of an IR-based facial-gesture tracking sensor and a corresponding sensor data classifier. The goal of the proposed technique was to provide a mouse interface-like simple, robust, and cost-effective UI for AR-based assistance. A wink gesture of the headset wearer was captured as an input command by a custom-designed skin-deformation-detection sensor. We implemented an unsupervised learning-based sensor data classifier network to classify the sensor signal. We collected 81,758 IR images for training of the proposed classifier network, and the network achieved 95.4% user-intention-detection accuracy in the proposed UI test application.

## Figures and Tables

**Table 1:** Real-time measurement results of gesture detection accuracy using the proposed AR headset.

Participant IDs	1	2	3	4	5	6	7	8	9	10
The Number of detected gestures	100	100	100	100	100	100	100	100	100	100
The Number of intended gestures	104	110	102	107	111	101	100	103	101	109
Accuracy of gesture recognition (%)	96.2	91.0	98.0	93.5	90.1	99.0	100	97.1	99.0	91.7
Clustering entropy	0.12	0.23	0.07	0.18	0.24	0.04	0	0.10	0.04	0.21
